# COVID-related healthcare disruptions among older adults with multiple chronic conditions in New York City

**DOI:** 10.1186/s12913-024-12114-5

**Published:** 2025-03-05

**Authors:** Lorna E. Thorpe, Yuchen Meng, Sarah Conderino, Samrachana Adhikari, Stefanie Bendik, Mark Weiner, Cathy Rabin, Melissa Lee, Jenny Uguru, Jasmin Divers, Annie George, John A. Dodson

**Affiliations:** 1 Department of Population Health, NYU Grossman School of Medicine, 180 Madison Avenue 9th Floor, New York, NY 10016 USA; 2 Department of Medicine, NYU Grossman School of Medicine, New York, NY USA; 3 Department of Foundations of Medicine, NYU Long Island School of Medicine, New York, NY USA; 4 INSIGHT Clinical Research Network, New York, NY USA; 5 NYC Health + Hospitals, New York, NY USA

**Keywords:** COVID-19, Healthcare utilization, Older adults, Multiple chronic conditions, Care disruption, New York City

## Abstract

**Background:**

Results from national surveys indicate that many older adults reported delayed medical care during the acute phase of the COVID-19 pandemic, yet few studies have used objective data to characterize healthcare utilization among vulnerable older adults in that period. In this study, we characterized healthcare utilization during the acute pandemic phase (March 7–October 6, 2020) and examined risk factors for total disruption of care among older adults with multiple chronic conditions (MCC) in New York City.

**Methods:**

This retrospective cohort study used electronic health record data from NYC patients aged ≥ 50 years with a diagnosis of either hypertension or diabetes and at least one other chronic condition seen within six months prior to pandemic onset and after the acute pandemic period at one of several major academic medical centers contributing to the NYC INSIGHT clinical research network (*n*=276,383). We characterized patients by baseline (pre-pandemic) health status using cutoffs of systolic blood pressure (SBP) < 140mmHg and hemoglobin A1C (HbA1c) < 8.0% as: controlled (below both cutoffs), moderately uncontrolled (below one), or poorly controlled (above both, SBP > 160, HbA1C > 9.0%). Patients were then assessed for total disruption versus some care during shutdown using recommended care schedules per baseline health status. We identified independent predictors for total disruption using logistic regression, including age, sex, race/ethnicity, baseline health status, neighborhood poverty, COVID infection, number of chronic conditions, and quartile of prior healthcare visits.

**Results:**

Among patients, 52.9% were categorized as controlled at baseline, 31.4% moderately uncontrolled, and 15.7% poorly controlled. Patients with poor baseline control were more likely to be older, female, non-white and from higher poverty neighborhoods than controlled patients (*P* < 0.001). Having fewer pre-pandemic healthcare visits was associated with total disruption during the acute pandemic period (adjusted odds ratio [aOR], 8.61, 95% Confidence Interval [CI], 8.30-8.93, comparing lowest to highest quartile). Other predictors of total disruption included self-reported Asian race, and older age.

**Conclusions:**

This study identified patient groups at elevated risk for care disruption. Targeted outreach strategies during crises using prior healthcare utilization patterns and disease management measures from disease registries may improve care continuity.

**Supplementary Information:**

The online version contains supplementary material available at 10.1186/s12913-024-12114-5.

## Introduction

In the most acute phase of the SARS-CoV-2 (COVID-19) pandemic, healthcare utilization patterns were dramatically altered across the United States, both due to health systems formally pausing outpatient visits and elective procedures, as well as patients choosing isolation to reduce risk of infection. A small number of published studies have characterized disruptions in healthcare in various patient populations [1], including disruptions in the public, among Veterans [2, 3], or among patients with specific heath conditions, such as patients with diabetes or serious mental illness [4–6]. However, little is known about the extent of disruption that occurred among older adults with multiple chronic conditions (MCC). During the early pandemic, older people with MCC including diabetes, hypertension, obesity, dyslipidemia, and chronic kidney disease–conditions which are increasingly common with age–were left extremely vulnerable to disruptions in healthcare delivery [7]. The recommended frequency of ambulatory care visits for a typical patient with MCC varies depending on number and stability of chronic conditions they have, ranging from every 3–6 months for more stable patients to more frequent visits (e.g. monthly) for patients with poorly controlled cardiovascular disease (CVD) risk factors.

In New York City (NYC), the first major U.S. metropolitan area affected by the COVID-19 pandemic, the first SARS CoV-2 case was identified on March 1, 2020 [8]; within 2 weeks, all elective medical procedures were canceled, and most ambulatory care practices closed their doors entirely [9]. Traditional ambulatory care ceased entirely for several months and then reopened at only limited capacity [10]. The shutdown of all but emergency care at many health systems across New York City prevented access to primary and other forms of ambulatory care for several months. Many patients with chronic medical conditions were left without a usual source of care. Several chronic medical conditions require frequent visits when poorly controlled, which was suddenly no longer feasible. While health systems attempted to bridge this gap through telemedicine and modified in-person visits, these strategies were adopted unevenly.

In this study, we sought to examine longitudinal trends in ambulatory care visits during the first year of the pandemic among NYC older adults, restricted to those with two or more diagnosed chronic conditions. Using electronic health record (EHR) data from a large clinical research network composed of several major academic medical centers, we quantified the extent to which these patients had disrupted care, using durations of recommended care identified based on their disease status immediately prior to the shutdown. This detailed descriptive analysis of healthcare utilization is a critical step towards examining the impacts of the pandemic-associated shutdown on cardiovascular health among people with MCC.

## Methods

### Data sources

This study used large-scale, de-identified EHR data from the INSIGHT Clinical Research Network (CRN). INSIGHT is the largest urban CRN in the US, capturing EHR data on more than 19 million diverse patients from seven academic healthcare systems in New York, NY and Houston, TX (Weill Cornell Medicine, Columbia University, Albert Einstein School of Medicine/Montefiore Medical Center, lcahn School of Medicine/Mount Sinai Health System, New York University Langone Health, New York-Presbyterian Hospital, and Houston Methodist). Houston Methodist data was not included in this analysis, as our interest was to characterize disruption among NYC residents. Zip code-level poverty data in 2019, derived from the American Community Survey Zip Code Tabulation Area (ZCTA) tables, was linked to EHR data based on residential five-digit zip codes to acquire zip code-level poverty characteristics.

### Study population

The eligible population included patients aged 50 and older who resided in NYC, were seen at facilities contributing EHR data to NYC INSIGHT clinical research network, and who had a hypertension or diabetes diagnosis and at least one other diagnosed chronic condition prior to the acute pandemic-associated healthcare shutdown. Specific chronic conditions were derived from the Department of Health and Human Services Initiative on multiple chronic conditions and enumerated. These included arthritis, asthma, atrial fibrillation, breast cancer, colorectal cancer, lung cancer, prostate cancer, chronic kidney disease, chronic obstructive pulmonary disease, depression, diabetes, heart failure, hyperlipidemia, hypertension, ischemic heart disease, osteoporosis, and stroke [11] (see Appendix A for ICD-10 codes).

To minimize pre-pandemic mortality and ensure that baseline information was accurate of recent health status, we restricted analyses to adults who had at least one documented ambulatory care visit within 6 months prior to acute pandemic onset, from September 7, 2019 to March 7, 2020. To minimize the effects of attrition from mortality or other loss-to-follow-up in the INSIGHT network, we further restricted the sample to individuals with at least one documented visit following the acute pandemic period. We excluded 20,504 (7.0%) patients who were missing both HbA1c and SBP measurements during that window and thus unable to be characterized in terms of baseline control status. Individuals with diagnosis of metastatic cancer, as well as Alzheimer’s disease or other form of dementia, were excluded from the study cohort given different preventative goals in these populations (see Study Population Flowchart, Appendix B).

### Ambulatory care disruption

**Fig. 1:**
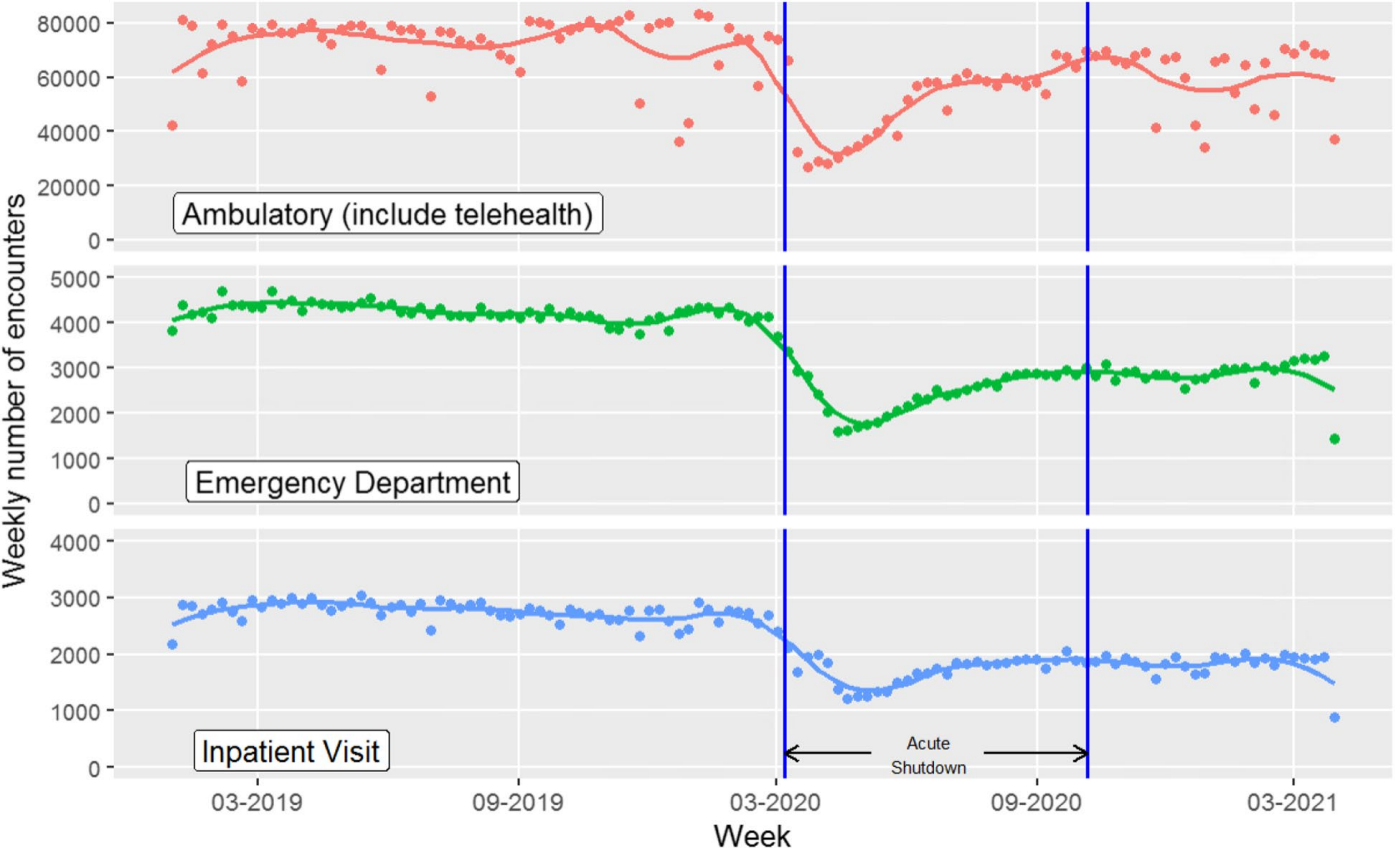
Characterization of weekly ambulatory, ED and Inpatient encounters among INSIGHT patients, NYC: January 2019-April 2021. *Acute Shutdown period, March 7-Oct 7, 2020

The primary outcome for this analysis was healthcare utilization disruption during the acute pandemic period. We defined this period to be March 7, 2020 through October 6, 2020, reflecting the observed acute shutdown of elective procedures and ambulatory care services through July 9, 2020 and the immediate recovery period that followed, through October 6, 2020. (Fig. 1). No clinical recommendations exist to guide the recommended frequency of follow up care for the full spectrum of predominantly older patients with MCC, but up-to-date clinical guidelines for blood pressure and diabetes [12, 13], as well as gerontology literature [14] broadly categorize recommended care intervals based on patients’ stability of risk factors such systolic blood pressure and hemoglobin A1c, the two major CVD risk factors that most frequently require treatment titration during ambulatory visits. In most circumstances, older patients with MCC should be seen at a minimum of 6-month intervals. Thus, no care during the 7-month acute period was defined as ‘total disruption’, or no care between March 7, 2020 and October 6, 2020. To descriptively assess whether total care disruption during the pandemic was elevated above pre-pandemic times, we characterized baseline levels of disruption in care using two definitions: (1) no care in the equivalent 7-month period of the prior year (March 7, 2019 – October 6, 2019) and (2) no care in the immediate 7 months prior to the last pre-pandemic visit.

Baseline health status was defined as either controlled, moderately uncontrolled or poorly controlled based on laboratory values from the last two ambulatory care visits prior to the acute pandemic onset. Controlled patients had SBP consistently below 140 mmHg and HbA1c consistently below 8.0% at last two visits prior to March 7, 2020. Moderately uncontrolled patients had either at least one SBP that was 140–159 mmHg or at least one HbA1c that was 8.0–8.9% at last two visits prior to March 7, 2020. Poorly controlled patients had (1) at least one SBP higher than 140 mmHg and at least one HbA1c higher than 8.0%, (2) SBP higher than 160 mmHg, or (3) HbA1c higher than 9.0%. For patients with multiple same-day SBP or HbA1c measurements, the lowest measurements in a day were used.

### Covariates

For descriptive analyses, age was categorized into four groups: 50–64, 65–74, 75–84 and over 85. Sex was coded into three categories: male, female, and unknown. Race and ethnicity were combined and categorized as Hispanic, Black, White, Asian, Other or Unknown. While individual patient baseline socioeconomic status (SES) was unknown, patients were categorized according to percent of neighborhood (Zip Code-level) residents living below the federal poverty level, divided into four categories: low (< 10%), medium (10- < 20%), high (20- < 30%) and very high (≥ 30%) [15].

Patients were also categorized according to the number of diagnosed chronic conditions they had prior to the pandemic and divided into four groups: 2, 3, 4 and 5 or more conditions. Patient healthcare utilization patterns prior to the pandemic were defined as the total number of ambulatory encounters in the prior four years, from March 7, 2016 through March 6, 2020, which were then categorized into quartiles (0–9, 9–18, 18–32 and more than 32 encounters). Having COVID-19 could result in a healthcare encounter during the period of interest and was defined based on presence of a COVID-19 diagnosis or positive lab result prior to end of acute pandemic period (October 6, 2020).

Ambulatory encounters during the study period of interest were defined as either in-person or telehealth. Telehealth encounters included telemedicine or virtual visits, which were conducted via video or phone. In-person encounters included visits at outpatient clinics, physician offices, same day/ambulatory surgery centers, urgent care facilities, and other same-day ambulatory hospital encounters, but excluded emergency department encounters, hospice visits, rehabilitation visits, home health visits, skilled nursing visits, other non-hospital visits and email consultations.

### Statistical analysis

We summarized patient characteristics and level of care disruption using descriptive statistics for the full sample and stratified by baseline (pre-pandemic) health status. To identify independent predictors of care disruption, classified as total disruption versus some care, we used binary logistic regression to examine univariate associations, demographic-adjusted associations, controlling for age group, sex, race/ethnicity, and neighborhood SES, and finally fully adjusted associations, controlling for demographics, prior health status, pre-pandemic healthcare utilization, number of chronic conditions, and COVID-19 infection. Patients with missing neighborhood SES data (*n* = 54) or unknown sex (*n* = 41) were excluded from regression analyses.

In secondary analyses (supplemental tables), we explored differential patterns in care disruption by running binary logistic regression models stratified by prior health status. We also examined the demographic and health status characteristics of NYC INSIGHT patients with MCC by the type of ambulatory care visits they had (in-person only, in-person and telehealth, telehealth only, no visits). Last, we examined predictors of disruption during the acute pandemic period among patients with no evidence of disruption in either of the pre-pandemic comparison periods. All data analyses were performed using SAS version 9.4 and R Studio version 4.2.1. Mean values are reported with standard deviations and regression exponentiated coefficients are reported as odds ratios with corresponding 95% confidence intervals computed as a Wald interval. Statistical significance was set at *P* < 0.05, and all tests were two-tailed unadjusted for multiple testing.

## Results

### Baseline characteristics

Within the INSIGHT clinical research network, a total of 296,887 unique patients with hypertension or diabetes and at least one other chronic condition aged 50 and older residing in NYC had at least one ambulatory visit in the six-month window prior to onset of the pandemic (September 7, 2019–March 7, 2020) and at least one visit post the acute pandemic period. After excluding patients missing SBP and HbA1c measures, 276,383 patients remained in our study population. Of these, 35.1% patients were aged 50–64, 33.8% were 65–74, 22.7% were 75–84, and 8.4% patients were aged 85 and older (Table 1). More than half (58.6%) were female, 36.2% were white, 21.6% were Hispanic, 18.9% were black, and 17.6% were unknown. Two-thirds (65.8%) lived in low or medium-poverty neighborhoods. More than one-third (35.9%) of patients had 5 or more diagnosed chronic conditions.

**Table 1:** Demographic Characteristics of NYC Residents Aged 50+ with Multiple Chronic Conditions, by Pre-Pandemic Health Status

	**Pre-Pandemic Health Status**			
	**Total** (N=276383*)	**Controlled^** **(N=146136, 52.9%)**	**Moderately Uncontrolled**^ǂ^ **(N=86783, 31.4%)**	**Poorly Controlled**^ꝉ^ **(N =43464, 15.7%)**
**Age**				
50-64	96939 (35.1%)	53943 (36.9%)	28131 (32.4%)	14865 (34.2%)
65-74	93520 (33.8%)	50053 (34.3%)	29530 (34.0%)	13937 (32.1%)
75-84	62644 (22.7%)	31180 (21.3%)	21170 (24.4%)	10294 (23.7%)
85+	23280 (8.4%)	10960 (7.5%)	7952 (9.2%)	4368 (10.1%)
**Sex**				
Male	114398 (41.4%)	61632 (42.2%)	35350 (40.7%)	17416 (40.1%)
Female	161944 (58.6%)	84483 (57.8%)	51423 (59.3%)	26038 (59.9%)
Unknown	41 (<0.1%)	21 (<0.1%)	10 (<0.1%)	10 (<0.1%)
**Race/Ethnicity**				
Asian	9943 (3.6%)	5978 (4.1%)	2762 (3.2%)	1203 (2.8%)
Black	52185 (18.9%)	23414 (16.0%)	17728 (20.4%)	11043 (25.4%)
Hispanic	59832 (21.6%)	30041 (20.6%)	18987 (21.9%)	10804 (24.9%)
White	100068 (36.2%)	58243 (39.9%)	30264 (34.9%)	11561 (26.6%)
Other	5790 (2.1%)	2944 (2.0%)	1803 (2.1%)	1043 (2.4%)
Unknown	48565 (17.6%)	25516 (17.5%)	15239 (17.6%)	7810 (18.0%)
**Neighborhood Poverty**				
Low	61589 (22.3%)	35880 (24.5%)	18303 (21.1%)	7406 (17.0%)
Medium	120331 (43.5%)	64214 (43.9%)	37780 (43.5%)	18337 (42.2%)
High	55938 (20.2%)	27831 (19.0%)	18114 (20.9%)	9993 (23.0%)
Very High	38471 (13.9%)	18173 (12.4%)	12575 (14.5%)	7723 (17.8%)
Unknown	54 (<0.1%)	38 (<0.1%)	11 (<0.1%)	5 (<0.1%)
**Number of prepandemic healthcare visits**				
Quantile 1**	60994 (22.1%)	33183 (22.7%)	18689 (21.5%)	9122 (21.0%)
Quantile 2	69544 (25.2%)	37327 (25.5%)	21769 (25.1%)	10448 (24.1%)
Quantile 3	68114 (24.6%)	35563 (24.3%)	21802 (25.1%)	10749 (24.7%)
Quantile 4	77731 (28.1%)	40063 (27.4%)	24523 (28.3%)	13145 (30.2%)
**Number of chronic conditions**				
2	50816 (18.4%)	27604 (18.9%)	16487 (19.0%)	6725 (15.5%)
3	66904 (24.2%)	36350 (24.9%)	21044 (24.2%)	9510 (21.9%)
4	59346 (21.5%)	31537 (21.6%)	18479 (21.3%)	9330 (21.5%)
5+	99317 (35.9%)	50645 (34.7%)	30773 (35.5%)	17899 (41.2%)
**Pre-pandemic Diagnoses**				
Arthritis	112573 (40.7%)	59325 (40.6%)	36393 (41.9%)	16865 (38.8%)
Asthma	41951 (15.2%)	21955 (15.0%)	13259 (15.3%)	6737 (15.5%)
Atrial fibrilation	51480 (18.6%)	28909 (19.8%)	15611 (18.0%)	6950 (16.0%)
Cancer***	30011 (10.9%)	14996 (10.3%)	10196 (11.7%)	4819 (11.1%)
CKD	55284 (20.0%)	24853 (17.0%)	17589 (20.2%)	12842 (29.5%)
COPD	39768 (14.4%)	21363 (14.6%)	12312 (14.2%)	6093 (14.0%)
Depression	46133 (16.7%)	24598 (16.8%)	14179 (16.3%)	7356 (16.9%)
Diabetes	121430 (43.9%)	58926 (40.3%)	36280 (41.8%)	26224 (60.3%)
Heart failure	32980 (11.9%)	17475 (12.0%)	9605 (11.1%)	5900 (13.6%)
Hyperlipidemia	209593 (75.8%)	111906 (76.6%)	64837 (74.7%)	32850 (75.6%)
Hypertension	262125 (94.8%)	136651 (93.5%)	83740 (96.5%)	41734 (96.0%)
IHD	83339 (30.2%)	45188 (30.9%)	25157 (29.0%)	12994 (29.9%)
Osteoporosis	32535 (11.8%)	17589 (12.0%)	10446 (12.0%)	4500 (10.4%)
Stroke	21228 (7.7%)	10830 (7.4%)	6654 (7.7%)	3744 (8.6%)
**Hospitalizations**				
No	234532 (84.9%)	125320 (85.8%)	73602 (84.8%)	35610 (81.9%)
Yes	41851 (15.1%)	20816 (14.2%)	13181 (15.2%)	7854 (18.1%)
**ED visits**				
No	212032 (76.7%)	114301 (78.2%)	66394 (76.5%)	31337 (72.1%)
Yes	64351 (23.3%)	31835 (21.8%)	20389 (23.5%)	12127 (27.9%)
**Hospitalization & ED**				
Neither	194805 (70.5%)	105341 (72.1%)	60881 (70.2%)	28583 (65.8%)
Inpatient only	17227 (6.2%)	8960 (6.1%)	5513 (6.4%)	2754 (6.3%)
ED only	39727 (14.4%)	19979 (13.7%)	12721 (14.7%)	7027 (16.2%)
Both	24624 (8.9%)	11856 (8.1%)	7668 (8.8%)	5100 (11.7%)

[Abbreviations: ADRD = Alzheimer's and related dementias, CKD = Chronic kidney disease, COPD = Chronic obstructive pulmonary disease, IHD = Ischemic heart disease, ED=Emergency Department]

* Total: Patients missing both A1c & SBP measures were excluded (*n*=20,504)

^ Controlled: Systolic blood pressure (SBP) below 140 mmHg and HbA1c lower than 8.0% in last two visits prior to March 7, 2020

ǂ Moderately Uncontrolled: At least one SBP between 140-159 mmHg or at least one HbA1c between 8.0-8.9% in last two visits prior to March 7, 2020

ꝉ Poorly Controlled: Either (1) SBP above 140 mmHg and HbA1c higher than 8.0%, or (2) SBP higher than 160 mmHg, or (3) HbA1c higher than 9.0%

** Quantile 1: pre-pandemic utilization ≤9 encounters; quantile 2: pre-pandemic utilization between 10-18 visits; quantile 3: pre-pandemic utilization between 19-32 visits; quantile 4: pre-pandemic utilization >32 visits

*** Cancer = breast, colorectal, lung, prostate

In terms of clinical status immediately prior to the pandemic onset, more than half of patients (146136 or 52.9%) were characterized as having their blood pressure and HbA1C well-controlled, 86783 (31.4%) were moderately uncontrolled, and 43464 (15.7%) were poorly controlled (Table 1). Baseline control status groups varied by age group, race/ethnicity, and neighborhood poverty. Patients with controlled chronic conditions were more likely to be younger, male, white, and from low poverty neighborhoods. In contrast, patients whose chronic conditions were poorly controlled prior to the shutdown were more likely to be older, female, black or Hispanic, and have 5 or more chronic conditions.

### Ambulatory care disruption

**Fig. 2:**
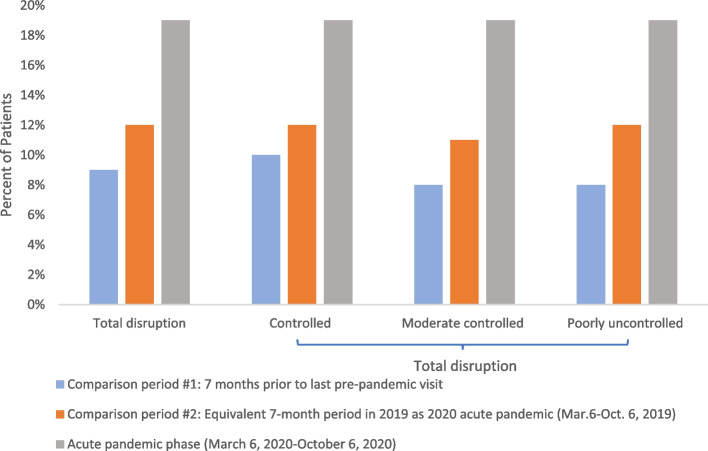
Comparison of patients experiencing care disruption during acute pandemic to two comparison time periods, overall and by pre-pandemic health status among NYC residents aged 50 + with MCC

As shown in Fig. 1, the overall number of ambulatory encounters, Emergency Department (ED) visits and inpatient visits dropped by more than half during the acute pandemic period between March and October, 2020, then rebounded but did not return to pre-pandemic levels for the rest of the year. During that acute period, approximately 19% of older patients with MCC experienced a total disruption of ambulatory care, and this proportion was consistent across groups defined by their baseline health status. Total disruption levels were higher during the pandemic time period than observed across two pre-pandemic comparison time periods, irrespective of baseline health status (Fig. 2). Outside of ambulatory care, a total of 29.5% of patients had either a hospitalization or an ED visit during the acute shutdown period (Table 1). Patients with poorly controlled chronic conditions at baseline were more likely to have a hospitalization or ED visit during the acute shutdown than well controlled patients (34.2% vs 27.9%).

**Fig. 3:**
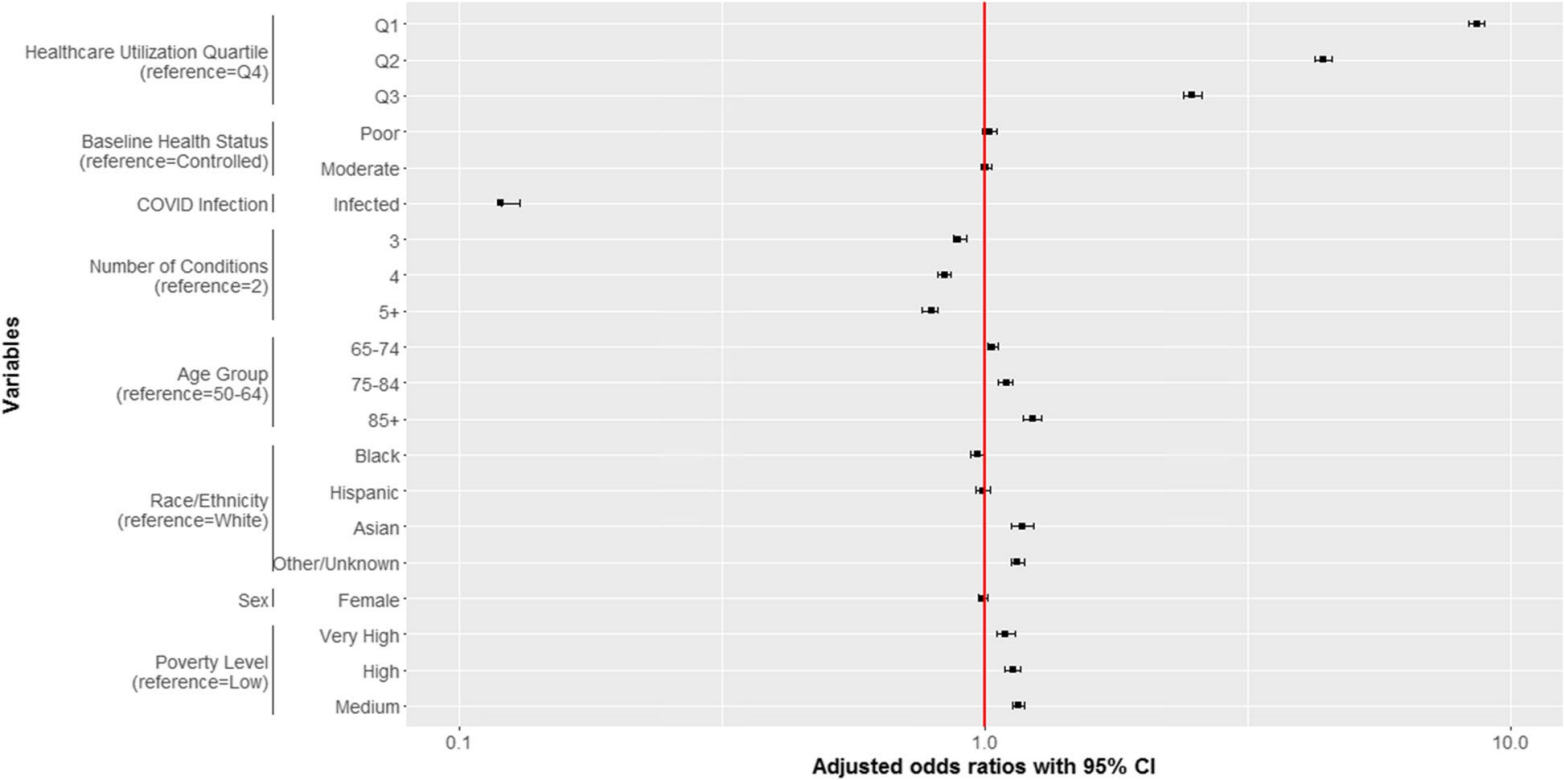
Predictors of Total Disruption During Acute Pandemic Phase (March 7, 2020 through October 6, 2020) among NYC Residents Aged 50 + with Multiple Chronic Conditions

In a multivariable model (Fig. 3), older patients and Asian patients had elevated odds of total disruption. In contrast, Black patients—and to a less extent Hispanic patients—were less likely than White patients to experience total disruption. The strongest predictors of total disruption were low prior healthcare utilization, older age (85 +), being of Asian ethnicity, and living in a higher poverty neighborhood. For example, patients who had infrequent visits prior to the pandemic were 8.6 times more likely to have total disruption compared to patients in the highest quartile of healthcare utilization (aOR 8.61, 95% CI 8.30, 8.93). Associations between neighborhood poverty level and total disruption were strong; after multivariable adjustment patients living in higher poverty regions were more likely to have total disruption than adults living in low poverty neighborhoods (aOR, 1.09, 95% CI, 1.05, 1.14, comparing very high poverty to low poverty). Patients with more chronic conditions were less likely to experience total disruption than patients with fewer conditions. (*P* < 0.01).

### Secondary analyses

In secondary analyses, we found that the magnitude of total ambulatory care disruption increased when telehealth encounters were excluded (from 19.1% to 28.4%). In total, 37.9% of patients had in-person visits only; 33.7% of patients had both in-person visits and telehealth encounters; and 9.3% of patients had only telehealth encounters. White patients were more likely to have in-person visits only, while younger patients (age 50–64), Hispanic patients and patients in higher poverty level neighborhoods were more likely to have telehealth-only visits. Patients with infrequent visits prior to the pandemic and fewer diagnosed chronic conditions were less likely to use in-person visits, while patients with frequent prior visits and more than 5 diagnosed chronic conditions were more likely to use in-person visits (Supplemental Table 1).

We also sought to identify subgroups of patients at risk for total disruption within each category of pre-pandemic health status by running stratified regression models within each group (Supplemental Table 2). Irrespective of level of baseline health status, patients in older age groups were more likely to have had total disruption than patients in younger age groups (*P* < 0.001). Similarly, in each of the groups, patients with fewer chronic conditions, higher pre-pandemic healthcare utilization, and who did not have a documented COVID-19 infection during the shutdown period all had elevated likelihood of total care disruption. Patterns of association between race/ethnicity and total disruption varied across the three stratified models. Among well-controlled and moderately uncontrolled patients, Asian patients were more likely than other race/ethnic groups to experience total disruption. Among moderately uncontrolled patients, we observed that Black patients had a lower likelihood of total disruption compared to other race/ethnic groups (aOR, 0.96, 95% CI, 0.91, 1.01). Last, among poorly controlled patients, we observed no differences in association with total disruption between Asian and white patients, but the protective association with Black patients was even more pronounced.

Finally, we ran a sensitivity analysis to account for patterns of care continuity prior to the pandemic. In this analysis, we examined predictors of total disruption in care during the pandemic *only* among those (*n* = 276,288) who had at least one encounter in the seven months prior to the last pre-pandemic encounter and at least one encounter in the equivalent seven-month period in 2019 (our two comparison time periods). While magnitudes of associations changed (as expected), predictors of disruption in this subset were comparable to the primary model depicted in Supplemental Table 3.

## Discussion

In this large study of ambulatory care patterns among more than 250,000 older adults with multiple chronic conditions living in NYC, we identified that one in five (19%) patients had no ambulatory care visits during the acute pandemic, per recommended care guidelines based on their categorized baseline health status. While telehealth visits played an important role in delivering care at a distance for this population–nearly 43% of all patients had at least one telehealth visit logged during that time—it only partially alleviated healthcare visit needs. These findings, together with the identification of clear predictors of patient subgroups at risk of total disruption, have important clinical and public health implications. In future crises, targeted outreach should be directed towards groups at high risk for not receiving care, including patients from high poverty neighborhoods, those with documented poor control of cardiovascular risk factors, older age groups, and patients with a documented history of infrequent healthcare utilization.

Our estimate that many NYC older adults with MCC may have experienced major care disruption during the acute pandemic period is consistent with a small number of studies that surveyed older adults in the United States about their experiences with delayed medical care [16–18]. Results from a one-time COVID-19 supplement questionnaire sent to older adult participants (aged 70 and older) in the National Health and Aging Trends Study (NHATS) indicated that 32% of older adults reported delayed care, with higher delays among adults who reported fair or poor self-reported health [16]. Similarly, according to a COVID-19 sub-study conducted among the National Social Life, Health and Aging Project (NSHAP), an ongoing nationally representative longitudinal study of U.S. community-dwelling older adults, one third of respondents (32.8%) indicated having delayed care during roughly the same period as studied in this manuscript [17]. In that representative sample, respondents with two or more chronic conditions in that study reported a higher odds of delayed care than adults with no chronic conditions (aOR 1.46, 95% CI 1.04–2.04). Finally, a third COVID-19 sub-study was conducted as part of the Health and Retirement Study (HRS). Estimates of delayed care were remarkably like the other two national studies—30% of U.S. older adults reported delayed care, with higher estimates among participants with poor self-reported health [18]. While in our study, we could not discern whether individual patients perceived their care to be insufficient, these national studies corroborate the fact that older patients with MCC reported high levels of delayed care.

Our objective measures of telehealth utilization indicate that many NYC older adults with MCC were able to obtain some or part of their care through telehealth visits, confirming that telehealth played an important role in sustaining care during the shutdown and initial recovery months. Indeed, many barriers to using telehealth were rapidly dismantled in the early weeks and months of the COVID-19 pandemic [19]. According to results published from a COVID-19 supplement to the nationally representative Medicare Current Beneficiary Survey of community-dwelling Medicare beneficiaries aged ≥ 65, 84% of participants reported their regular providers offered telehealth services during the early months of the COVID-19 pandemic [20]. In that study, 43% reported using telehealth services, an estimate remarkably similar to our measure of 43% telehealth use. This survey also found—like our study–that Hispanic and Black beneficiaries were more likely to report using telehealth services when offered compared to Non-Hispanic White beneficiaries. This may plausibly reflect that providers were differentially offering telehealth to lower-income or minority patients, as lower income subgroups of beneficiaries surveyed nationally have reported being offered telehealth more frequently than higher income subgroups [20]. Irrespective of whether such findings reflect the targeting of telehealth services or self-selected use, they offer some evidence that telehealth, when offered, may help to reduce disparities in care for some populations with historic care barriers. This higher uptake of telehealth among Hispanic older adults with MCC in NYC may partially explain the lower overall levels of disruption in care measured in our study.

This study has several potential limitations. Perhaps most important, this study only included patients who had recently received care at one of the facilities contributing to the NYC INSIGHT network, therefore results may not be generalizable to individuals who sought care in public hospital systems or who had not received care in the six months prior to pandemic onset. It is also plausible that some healthcare visits may have occurred outside of this network of NYC-area hospitals, resulting in missing data. Additionally, we characterized baseline (pre-pandemic) health status, as well as recommended periodicity of follow-up care, based on level of control of two CVD risk factors, systolic blood pressure and hemoglobin A1c. Given the heterogeneity of diagnosed chronic conditions experienced by patients in this large sample, it is highly plausible that some participants had poor health status requiring frequent clinical care visits whose SBP and A1c were well controlled, and in that sense were misclassified in both their health status and recommended care schedule. However, given the near ubiquity of cardiometabolic conditions in this sample, the frequency with which BP and A1c are measured during ambulatory care visits, and the lack of other standardized or overarching measures of health status, we believe these to be the most appropriate and well-measured indicators routinely available in EHR data to assess care disruption within strata of baseline health status. Indeed, results were quite similar when we restricted our analyses to patients with diagnosed hypertension or diabetes. Approximately 18% of patients had missing race/ethnicity data, and unknown race/ethnicity was associated with total care disruption in our adjusted model, suggesting that race/ethnic-specific estimates could be biased if missingness clustered within specific race/ethnic subgroups. Finally, due to the lack of specificity in the EHR common data model used, we also were unable to disentangle the ambulatory care visits into primary care versus other forms of ambulatory care, and race/ethnicity had a high proportion of missing data. Nonetheless, the sharp concordance between our study and results from several nationally representative surveys of older adults suggests that the construction of our measure and findings are sufficiently valid to yield consistent results.

Despite these limitations our study has several key strengths, including a large sample size that used objective EHR data systematically extracted and harmonized from multiple major academic medical centers in the NYC area, which allowed us to systematically characterize care disruption among the vulnerable population subgroup of older adults with MCC. An additional strength was that we employed a novel measure of care disruption for this population, basing the recommended care schedule on their pre-pandemic health status, as measured by common CVD risk factors. Not only did this yield consistent findings compared with national surveys, but it also offers a model for stratification and assessing healthcare utilization trajectories for future EHR-based studies of patients with MCC.

In sum, our findings identified subgroups that were at highest risk for disruptions in care during the acute COVID-19 period and demonstrated the importance of telehealth in improving care continuity. These findings suggest that health care systems may need to employ multiple approaches to provide continuity of medical services to older adults who are most in need of care during future crises. Sustaining and expanding telehealth services is clearly an important strategy, but protocols could assess whether patients do not wish or cannot use telehealth. Most importantly, clinics can use prior healthcare utilization patterns and disease management measures from chronic disease registries to target outreach to patients at highest risk for total disruption.

## Supplementary Information

Supplementary Material 1. Supplemental Table 1. Demographic and Health Status Characteristics of NYC Residents Aged 50 and Older with Multiple Chronic Conditions Receiving Care at NYC INSIGHT Facilities by Type of Visit, September 2019-March 2020. Supplemental Table 2. Demographic and Clinical Predictors of Total Disruption During the Acute Pandemic Stratified by Pre-Pandemic Control Status among NYC Residents Aged 50 and Older with Multiple Chronic Conditions Receiving Care at NYC INSIGHT Facilities, September 2019-March 2020. Supplemental Table 3. Demographic and Clinical Predictors of Total Disruption During the Acute Pandemic among NYC Residents Aged 50 and Older with Multiple Chronic Conditions and with Hypertension or Diabetes, NYC INSIGHT Facilities, September 2019-March 2020.

## Figures and Tables

**Figure:**
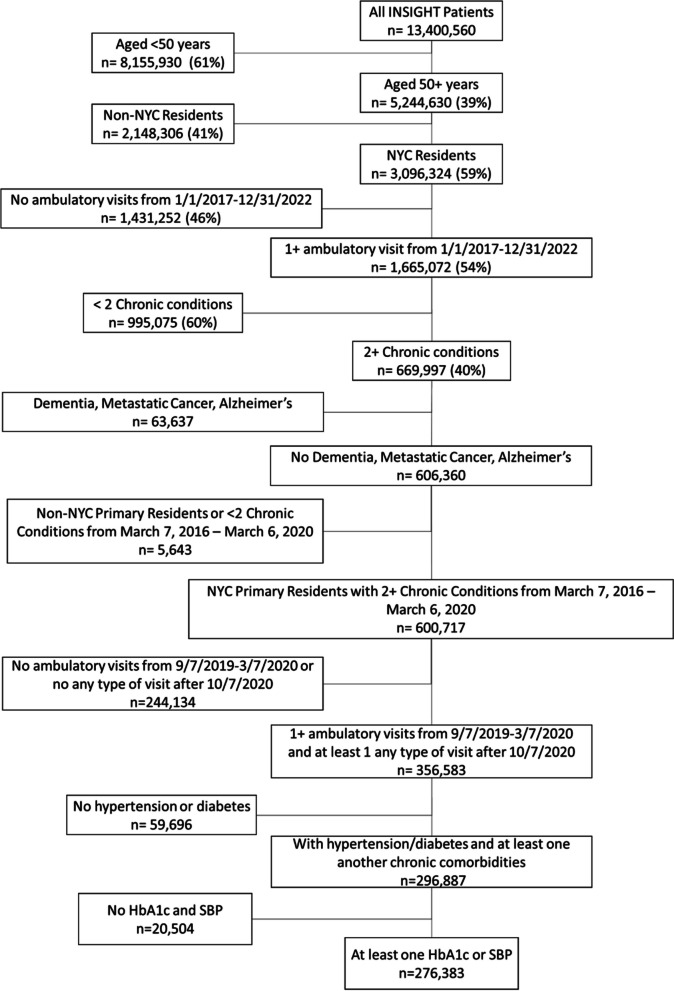
INSIGHT flow chart

## Data Availability

The data that support the findings of this study are available from INSIGHT Clinical Research Network but restrictions apply to the availability of these data, which were used under license for the current study and therefore are not publicly available. Data are available from the authors upon reasonable request and with permission of INSIGHT Clinical Research Network.

## Appendix A

**Table 2:** ICD-10 codes used to define comorbidities and exclusionary conditions

**Condition**	**ICD-10 CM Codes**
Arthritis	M0500, M05011, M05012, M05019, M05021, M05022, M05029, M05031, M05032, M05039, M05041, M05042, M05049, M05051, M05052, M05059, M05061, M05062, M05069, M05071, M05072, M05079, M0509, M0510, M05111, M05112, M05119, M05121, M05122, M05129, M05131, M05132, M05139, M05141, M05142, M05149, M05151, M05152, M05159, M05161, M05162, M05169, M05171, M05172, M05179, M0519, M0520, M05211, M05212, M05219, M05221, M05222, M05229, M05231, M05232, M05239, M05241, M05242, M05249, M05251, M05252, M05259, M05261, M05262, M05269, M05271, M05272, M05279, M0529, M0530, M05311, M05312, M05319, M05321, M05322, M05329, M05331, M05332, M05339, M05341, M05342, M05349, M05351, M05352, M05359, M05361, M05362, M05369, M05371, M05372, M05379, M0539, M0540, M05411, M05412, M05419, M05421, M05422, M05429, M05431, M05432, M05439, M05441, M05442, M05449, M05451, M05452, M05459, M05461, M05462, M05469, M05471, M05472, M05479, M0549, M0550, M05511, M05512, M05519, M05521, M05522, M05529, M05531, M05532, M05539, M05541, M05542, M05549, M05551, M05552, M05559, M05561, M05562, M05569, M05571, M05572, M05579, M0559, M0560, M05611, M05612, M05619, M05621, M05622, M05629, M05631, M05632, M05639, M05641, M05642, M05649, M05651, M05652, M05659, M05661, M05662, M05669, M05671, M05672, M05679, M0569, M0570, M05711, M05712, M05719, M05721, M05722, M05729, M05731, M05732, M05739, M05741, M05742, M05749, M05751, M05752, M05759, M05761, M05762, M05769, M05771, M05772, M05779, M0579, M057A, M0580, M05811, M05812, M05819, M05821, M05822, M05829, M05831, M05832, M05839, M05841, M05842, M05849, M05851, M05852, M05859, M05861, M05862, M05869, M05871, M05872, M05879, M0589, M058A, M059, M0600, M06011, M06012, M06019, M06021, M06022, M06029, M06031, M06032, M06039, M06041, M06042, M06049, M06051, M06052, M06059, M06061, M06062, M06069, M06071, M06072, M06079, M0608, M0609, M060A, M061, M0620, M06211, M06212, M06219, M06221, M06222, M06229, M06231, M06232, M06239, M06241, M06242, M06249, M06251, M06252, M06259, M06261, M06262, M06269, M06271, M06272, M06279, M0628, M0629, M0630, M06311, M06312, M06319, M06321, M06322, M06329, M06331, M06332, M06339, M06341, M06342, M06349, M06351, M06352, M06359, M06361, M06362, M06369, M06371, M06372, M06379, M0638, M0639, M064, M0680, M06811, M06812, M06819, M06821, M06822, M06829, M06831, M06832, M06839, M06841, M06842, M06849, M06851, M06852, M06859, M06861, M06862, M06869, M06871, M06872, M06879, M0688, M0689, M068A, M069, M0800, M08011, M08012, M08019, M08021, M08022, M08029, M08031, M08032, M08039, M08041, M08042, M08049, M08051, M08052, M08059, M08061, M08062, M08069, M08071, M08072, M08079, M0808, M0809, M080A, M081, M0820, M08211, M08212, M08219, M08221, M08222, M08229, M08231, M08232, M08239, M08241, M08242, M08249, M08251, M08252, M08259, M08261, M08262, M08269, M08271, M08272, M08279, M0828, M0829, M082A, M083, M0840, M08411, M08412, M08419, M08421, M08422, M08429, M08431, M08432, M08439, M08441, M08442, M08449, M08451, M08452, M08459, M08461, M08462, M08469, M08471, M08472, M08479, M0848, M084A, M0880, M08811, M08812, M08819, M08821, M08822, M08829, M08831, M08832, M08839, M08841, M08842, M08849, M08851, M08852, M08859, M08861, M08862, M08869, M08871, M08872, M08879, M0888, M0889, M0890, M08911, M08912, M08919, M08921, M08922, M08929, M08931, M08932, M08939, M08941, M08942, M08949, M08951, M08952, M08959, M08961, M08962, M08969, M08971, M08972, M08979, M0898, M0899, M089A, M150, M151, M152, M153, M154, M158, M159, M160, M1610, M1611, M1612, M162, M1630, M1631, M1632, M164, M1650, M1651, M1652, M166, M167, M169, M170, M1710, M1711, M1712, M172, M1730, M1731, M1732, M174, M175, M179, M180, M1810, M1811, M1812, M182, M1830, M1831, M1832, M184, M1850, M1851, M1852, M189, M19011, M19012, M19019, M19021, M19022, M19029, M19031, M19032, M19039, M19041, M19042, M19049, M19071, M19072, M19079, M1909, M19111, M19112, M19119, M19121, M19122, M19129, M19131, M19132, M19139, M19141, M19142, M19149, M19171, M19172, M19179, M1919, M19211, M19212, M19219, M19221, M19222, M19229, M19231, M19232, M19239, M19241, M19242, M19249, M19271, M19272, M19279, M1929, M1990, M1991, M1992, M1993, M450, M451, M452, M453, M454, M455, M456, M457, M458, M459, M45A0, M45A1, M45A2, M45A3, M45A4, M45A5, M45A6, M45A7, M45A8, M45AB, M488X1, M488X2, M488X3, M488X4, M488X5, M488X6, M488X7, M488X8, M488X9, L4050, L4051, L4054, L4059, M4680, M4681, M4682, M4683, M4684, M4685, M4686, M4687, M4688, M4689, M4690, M4691, M4692, M4693, M4694, M4695, M4696, M4697, M4698, M4699, M47011, M47012, M47013, M47014, M47015, M47016, M47019, M47021, M47022, M47029, M4710, M4711, M4712, M4713, M4714, M4715, M4716, M4720, M4721, M4722, M4723, M4724, M4725, M4726, M4727, M4728, M47811, M47812, M47813, M47814, M47815, M47816, M47817, M47818, M47819, M47891, M47892, M47893, M47894, M47895, M47896, M47897, M47898, M47899, M479
Asthma	J4520, J4521, J4522, J4530, J4531, J4532, J4540, J4541, J4542, J4550, J4551, J4552, J45901, J45902, J45909, J45990, J45991, J45998
Atrial Fibrillation	I470, I471, I472, I479, I480, I481, I4811, I4819, I482, I4820, I4821, I483, I484, I4891, I4892, I491, I492, I493, I4940, I4949, I495, I498, I499
Breast Cancer	C50011, C50012, C50019, C50021, C50022, C50029, C50111, C50112, C50119, C50121, C50122, C50129, C50211, C50212, C50219, C50221, C50222, C50229, C50311, C50312, C50319, C50321, C50322, C50329, C50411, C50412, C50419, C50421, C50422, C50429, C50511, C50512, C50519, C50521, C50522, C50529, C50611, C50612, C50619, C50621, C50622, C50629, C50811, C50812, C50819, C50821, C50822, C50829, C50911, C50912, C50919, C50921, C50922, C50929, D0500, D0501, D0502, D0510, D0511, D0512, D0580, D0581, D0582, D0590, D0591, D0592, Z170, Z171, Z191, Z192, Z853, Z86000
Chronic Kidney Disease	D631, E0822, E0922, E1022, E1122, E1322, I120, I129, I130, I1310, I1311, I132, N181, N182, N183, N1830, N1831, N1832, N184, N185, N186, N189, R880, Z4901, Z4902, Z4931, Z4932, A1811, A5275, B520, E0821, E0829, E0921, E0929, E1021, E1029, E1121, E1129, E1321, E1329, K767, M1030, M10311, M10312, M10319, M10321, M10322, M10329, M10331, M10332, M10339, M10341, M10342, M10349, M10351, M10352, M10359, M10361, M10362, M10369, M10371, M10372, M10379, M1038, M1039, M3214, M3215, M3504, M350A, N010, N011, N012, N013, N014, N015, N016, N017, N018, N019, N01A, N020, N021, N022, N023, N024, N025, N026, N027, N028, N029, N02A, N030, N031, N032, N033, N034, N035, N036, N037, N038, N039, N03A, N040, N041, N042, N043, N044, N045, N046, N047, N048, N049, N04A, N050, N051, N052, N053, N054, N055, N056, N057, N058, N059, N05A, N060, N061, N062, N063, N064, N065, N066, N067, N068, N069, N06A, N070, N071, N072, N073, N074, N075, N076, N077, N078, N079, N07A, N08, N140, N141, N142, N143, N144, N150, N158, N159, N16, N251, N2589, N259, N261, N269, N990, Q6102, Q6111, Q6119, Q612, Q613, Q614, Q615, Q618
Colorectal Cancer	C180, C181, C182, C183, C184, C185, C186, C187, C188, C189, C19, C20, C260, C49A4, C49A5, D010, D011, D012, D0140, Z85030, Z85038, Z85040, Z85048
COPD	J410, J411, J418, J42, J430, J431, J432, J438, J439, J440, J441, J449, J470, J471, J479, J40, J982, J983
Depression	F0631, F0632, F0634, F320, F321, F322, F323, F324, F328, F3281, F3289, F329, F32A, F330, F331, F332, F333, F3341, F338, F339, F341, F310, F3110, F3111, F3112, F3113, F312, F3130, F3131, F3132, F314, F315, F3160, F3161, F3162, F3163, F3164, F3171, F3173, F3175, F3176, F3177, F3178, F3181, F3189, F319, F325, F3340, F3342, F340, F4321, F4323
Diabetes	E0800, E0801, E0810, E0811, E0821, E0822, E0829, E08311, E08319, E08321, E083211, E083212, E083213, E083219, E08329, E083291, E083292, E083293, E083299, E08331, E083311, E083312, E083313, E083319, E08339, E083391, E083392, E083393, E083399, E08341, E083411, E083412, E083413, E083419, E08349, E083491, E083492, E083493, E083499, E08351, E083511, E083512, E083513, E083519, E083521, E083522, E083523, E083529, E083531, E083532, E083533, E083539, E083541, E083542, E083543, E083549, E083551, E083552, E083553, E083559, E08359, E083591, E083592, E083593, E083599, E0836, E0837X1, E0837X2, E0837X3, E0837X9, E0839, E0840, E0841, E0842, E0843, E0844, E0849, E0851, E0852, E0859, E08610, E08618, E08620, E08621, E08622, E08628, E08630, E08638, E08641, E08649, E0865, E0869, E088, E089, E0900, E0901, E0910, E0911, E0921, E0922, E0929, E09311, E09319, E09321, E093211, E093212, E093213, E093219, E09329, E093291, E093292, E093293, E093299, E09331, E093311, E093312, E093313, E093319, E09339, E093391, E093392, E093393, E093399, E09341, E093411, E093412, E093413, E093419, E09349, E093491, E093492, E093493, E093499, E09351, E093511, E093512, E093513, E093519, E093521, E093522, E093523, E093529, E093531, E093532, E093533, E093539, E093541, E093542, E093543, E093549, E093551, E093552, E093553, E093559, E09359, E093591, E093592, E093593, E093599, E0936, E0937X1, E0937X2, E0937X3, E0937X9, E0939, E0940, E0941, E0942, E0943, E0944, E0949, E0951, E0952, E0959, E09610, E09618, E09620, E09621, E09622, E09628, E09630, E09638, E09641, E09649, E0965, E0969, E098, E099, E1010, E1011, E1021, E1022, E1029, E10311, E10319, E10321, E103211, E103212, E103213, E103219, E10329, E103291, E103292, E103293, E103299, E10331, E103311, E103312, E103313, E103319, E10339, E103391, E103392, E103393, E103399, E10341, E103411, E103412, E103413, E103419, E10349, E103491, E103492, E103493, E103499, E10351, E103511, E103512, E103513, E103519, E103521, E103522, E103523, E103529, E103531, E103532, E103533, E103539, E103541, E103542, E103543, E103549, E103551, E103552, E103553, E103559, E10359, E103591, E103592, E103593, E103599, E1036, E1037X1, E1037X2, E1037X3, E1037X9, E1039, E1040, E1041, E1042, E1043, E1044, E1049, E1051, E1052, E1059, E10610, E10618, E10620, E10621, E10622, E10628, E10630, E10638, E10641, E10649, E1065, E1069, E108, E109, E1100, E1101, E1110, E1111, E1121, E1122, E1129, E11311, E11319, E11321, E113211, E113212, E113213, E113219, E11329, E113291, E113292, E113293, E113299, E11331, E113311, E113312, E113313, E113319, E11339, E113391, E113392, E113393, E113399, E11341, E113411, E113412, E113413, E113419, E11349, E113491, E113492, E113493, E113499, E11351, E113511, E113512, E113513, E113519, E113521, E113522, E113523, E113529, E113531, E113532, E113533, E113539, E113541, E113542, E113543, E113549, E113551, E113552, E113553, E113559, E11359, E113591, E113592, E113593, E113599, E1136, E1137X1, E1137X2, E1137X3, E1137X9, E1139, E1140, E1141, E1142, E1143, E1144, E1149, E1151, E1152, E1159, E11610, E11618, E11620, E11621, E11622, E11628, E11630, E11638, E11641, E11649, E1165, E1169, E118, E119, E1300, E1301, E1310, E1311, E1321, E1322, E1329, E13311, E13319, E13321, E133211, E133212, E133213, E133219, E13329, E133291, E133292, E133293, E133299, E13331, E133311, E133312, E133313, E133319, E13339, E133391, E133392, E133393, E133399, E13341, E133411, E133412, E133413, E133419, E13349, E133491, E133492, E133493, E133499, E13351, E133511, E133512, E133513, E133519, E133521, E133522, E133523, E133529, E133531, E133532, E133533, E133539, E133541, E133542, E133543, E133549, E133551, E133552, E133553, E133559, E13359, E133591, E133592, E133593, E133599, E1336, E1337X1, E1337X2, E1337X3, E1337X9, E1339, E1340, E1341, E1342, E1343, E1344, E1349, E1351, E1352, E1359, E13610, E13618, E13620, E13621, E13622, E13628, E13630, E13638, E13641, E13649, E1365, E1369, E138, E139, E891, O24011, O24012, O24013, O24019, O2402, O2403, O24111, O24112, O24113, O24119, O2412, O2413, O24311, O24312, O24313, O24319, O2432, O2433, O24410, O24414, O24415, O24419, O24420, O24424, O24425, O24429, O24430, O24434, O24435, O24439, O24811, O24812, O24813, O24819, O2482, O2483, O24911, O24912, O24913, O24919, O2492, O2493, O99810, O99814, O99815
Heart Failure	I0981, I110, I130, I132, I501, I5020, I5021, I5022, I5023, I5030, I5031, I5032, I5033, I5040, I5041, I5042, I5043, I50810, I50811, I50812, I50813, I50814, I5082, I5083, I5084, I5089, I509, I97130, I97131, O29121, O29122, O29123, O29129, Z95811, Z95812, I420, I425, I426, I427, I428, I43, P290
Hyperlipidemia	E780, E7800, E7801, E781, E782, E783, E784, E7841, E7849, E785
Hypertension	H35031, H35032, H35033, I10, I110, I119, I120, I129, I130, I1310, I1311, I132, I150, I151, I152, I158, I159, I160, I161, I169, I674, I868, I87001, I87002, I87003, I87009, I87011, I87012, I87013, I87019, I87021, I87022, I87023, I87029, I87031, I87032, I87033, I87039, I87091, I87092, I87093, I87099, I871, I872, I87301, I87302, I87303, I87309, I87311, I87312, I87313, I87319, I87321, I87322, I87323, I87329, I87331, I87332, I87333, I87339, I87391, I87392, I87393, I87399, I973, O10011, O10012, O10013, O10019, O1002, O1003, O10111, O10112, O10113, O10119, O1012, O1013, O10211, O10212, O10213, O10219, O1022, O1023, O10311, O10312, O10313, O10319, O1032, O1033, O10411, O10412, O10413, O10419, O1042, O1043, O10911, O10912, O10913, O10919, O1092, O1093, O111, O112, O113, O114, O115, O119, O131, O132, O133, O134, O135, O139, O161, O162, O163, O164, O165, O169, H35039, N262
Ischemic Heart Disease	I200, I201, I208, I209, I240, I248, I249, I2510, I25110, I25111, I25118, I25119, I252, I255, I256, I25700, I25701, I25708, I25709, I25710, I25711, I25718, I25719, I25720, I25721, I25728, I25729, I25730, I25731, I25738, I25739, I25750, I25751, I25758, I25759, I25760, I25761, I25768, I25769, I25790, I25791, I25798, I25799, I25810, I25811, I25812, I2582, I2583, I2584, I2589, I259, Z951, Z955, Z9861, I241, I253, I2541, I2542
Lung Cancer	C33, C3400, C3401, C3402, C3410, C3411, C3412, C342, C3430, C3431, C3432, C3480, C3481, C3482, C3490, C3491, C3492, C384, C390, C399, D021, D0220, D0221, D0222, D023, D024, Z85110, Z85118
Osteoporosis	M8000XA, M80011A, M80012A, M80019A, M80021A, M80022A, M80029A, M80031A, M80032A, M80039A, M80041A, M80042A, M80049A, M80051A, M80052A, M80059A, M80061A, M80062A, M80069A, M80071A, M80072A, M80079A, M8008XA, M800AXA, M8080XA, M80811A, M80812A, M80819A, M80821A, M80822A, M80829A, M80831A, M80832A, M80839A, M80841A, M80842A, M80849A, M80851A, M80852A, M80859A, M80861A, M80862A, M80869A, M80871A, M80872A, M80879A, M8088XA, M808AXA, M810, M816, M818
Prostate Cancer	C61, D075, Z8546
Stroke	I6000, I6001, I6002, I6010, I6011, I6012, I602, I6020, I6021, I6022, I6030, I6031, I6032, I604, I6050, I6051, I6052, I606, I607, I608, I609, I610, I611, I612, I613, I614, I615, I616, I618, I619, I6200, I6201, I6202, I6203, I621, I629, G450, G451, G452, G453, G458, G459, G460, G461, G462, G463, G464, G465, G466, G467, G468, G9731, G9732, I6300, I63011, I63012, I63013, I63019, I6302, I63031, I63032, I63033, I63039, I6309, I6310, I63111, I63112, I63113, I63119, I6312, I63131, I63132, I63133, I63139, I6319, I6320, I63211, I63212, I63213, I63219, I6322, I63231, I63232, I63233, I63239, I6329, I6330, I63311, I63312, I63313, I63319, I63321, I63322, I63323, I63329, I63331, I63332, I63333, I63339, I63341, I63342, I63343, I63349, I6339, I6340, I63411, I63412, I63413, I63419, I63421, I63422, I63423, I63429, I63431, I63432, I63433, I63439, I63441, I63442, I63443, I63449, I6349, I6350, I63511, I63512, I63513, I63519, I63521, I63522, I63523, I63529, I63531, I63532, I63533, I63539, I63541, I63542, I63543, I63549, I6359, I636, I638, I6381, I6389, I639, I67841, I67848, I6789, I97810, I97811, I97820, I97821
Alzheimer’s Disease	G300, G301, G308, G309
Dementia	F0150, F0151, F0280, F0281, F0390, F0391, F05, G138, G3101, G3109, G311, G312, G3183, G94, R4181
Metastatic Cancer	C770, C771, C772, C773, C774, C775, C778, C779, C7800, C7801, C7802, C781, C782, C7830, C7839, C784, C785, C786, C787, C7880, C7889, C7900, C7901, C7902, C7910, C7911, C7919, C792, C7931, C7932, C7940, C7949, C7951, C7952, C7960, C7961, C7962, C7970, C7971, C7972, C7981, C7982, C7989, C799

## Abbreviations

NYC: New York City
MCC: Multiple chronic conditions
SBP: Systolic blood pressure
HbA1c: Hemoglobin A1C
aOR: Adjusted odds ratio
CI: Confidence Interval
COVID-19: SARS-CoV-2
CVD: Cardiovascular disease
HER: Electronic health record
CRN: Clinical Research Network
ZCTA: Zip Code Tabulation Area
SES: Socioeconomic status
ED: Emergency Department
NHATS: National Health and Aging Trends Study
NSHAP: National Social Life, Health and Aging Project
HRS: Health and Retirement Study
ADRD: Alzheimer's and related dementias
CKD: Chronic kidney disease
COPD: Chronic obstructive pulmonary disease,
IHD: Ischemic heart disease
