# Supplementary material for: COVID-related healthcare disruptions among older adults with multiple chronic conditions in New York City
Source: BMC Health Serv Res. 2025 Mar 5;25:340. doi: 10.1186/s12913-024-12114-5 (PMC11881239; doi:10.1186/s12913-024-12114-5)
Supplement: Supplementary file 1 — Supplementary Material 1. Supplemental Table 1. Demographic and Health Status Characteristics of NYC Residents Aged 50 and Older with Multiple Chronic Conditions Receiving Care at NYC INSIGHT Facilities by Type of Visit, September 2019-March 2020. Supplemental Table 2. Demographic and Clinical Predictors of Total Disruption During the Acute Pandemic Stratified by Pre-Pandemic Control Status among NYC Residents Aged 50 and Older with Multiple Chronic Conditions Receiving Care at NYC INSIGHT Facilities, September 2019-March 2020. Supplemental Table 3. Demographic and Clinical Predictors of Total Disruption During the Acute Pandemic among NYC Residents Aged 50 and Older with Multiple Chronic Conditions and with Hypertension or Diabetes, NYC INSIGHT Facilities, September 2019-March 2020. [file 12913_2024_12114_MOESM1_ESM.docx]

**Supplement Table 1**

|  | **Type of Visit** | | | | | | | |
| --- | --- | --- | --- | --- | --- | --- | --- | --- |
|  | **Total**   **(N =276383*)** | | **In-person visit Only (N=104746, 37.9%)** | **In-person & Telehealth visit (N=93016, 33.7%)** | | **Telehealth visit Only (N=25762, 9.3%)** | | **Total disruption (N=52859, 19.1%)** |
| **Age** |  | |  |  | |  | |  |
| 50-64 | 96939 (35.1%) | | 31445 (30.0%) | 35330 (38.0%) | | **10960 (42.5%)** | | 19204 (36.3%) |
| 65-74 | 93520 (33.8%) | | 36165 (34.5%) | 31519 (33.9%) | | 8116 (31.5%) | | 17720 (33.5%) |
| 75-84 | 62644 (22.7%) | | 26797 (25.6%) | 19522 (21.0%) | | 4858 (18.9%) | | 11467 (21.7%) |
| 85+ | 23280 (8.4%) | | 10339 (9.9%) | 6645 (7.1%) | | 1828 (7.1%) | | 4468 (8.5%) |
| **Sex** |  | |  |  | |  | |  |
| Male | 114398 (41.4%) | | 45132 (43.1%) | 36006 (38.7%) | | 10297 (40.0%) | | 22963 (43.4%) |
| Female | 161944 (58.6%) | | 59600 (56.9%) | 56993 (61.3%) | | 15463 (60.0%) | | 29888 (56.6%) |
| Unknown | 41 (<0.1%) | | 14 (<0.1%) | 17 (<0.1%) | | 2 (<0.1%) | | 8 (<0.1%) |
| **Race/Ethnicity** |  | |  |  | |  | |  |
| Asian | 9943 (3.6%) | | 4124 (3.9%) | 2687 (2.9%) | | 786 (3.1%) | | 2346 (4.4%) |
| Black | 52185 (18.9%) | | 17154 (16.4%) | 20203 (21.7%) | | 5643 (21.9%) | | 9185 (17.4%) |
| Hispanic | 59832 (21.6%) | | 18965 (18.1%) | 23803 (25.6%) | | **6781 (26.3%)** | | 10283 (19.5%) |
| White | 100068 (36.2%) | | **45495 (43.4%)** | 28707 (30.9%) | | 6992 (27.1%) | | 18874 (35.7%) |
| Other | 5790 (2.1%) | | 1726 (1.6%) | 1920 (2.1%) | | 797 (3.1%) | | 1347 (2.5%) |
| Unknown | 48565 (17.6%) | | 17282 (16.5%) | 15696 (16.9%) | | 4763 (18.5%) | | 10824 (20.5%) |
| **Neighborhood Poverty** |  | |  |  | |  | |  |
| Low | 61589 (22.3%) | | 26655 (25.4%) | 18982 (20.4%) | | 4455 (17.3%) | | 11497 (21.8%) |
| Medium | 120331 (43.5%) | | 47657 (45.5%) | 37499 (40.3%) | | 10914 (42.4%) | | 24261 (45.9%) |
| High | 55938 (20.2%) | | 20266 (19.3%) | 19809 (21.3%) | | 5350 (20.8%) | | 10513 (19.9%) |
| Very High | 38471 (13.9%) | | 10148 (9.7%) | 16708 (18.0%) | | **5038 (19.6%)** | | 6577 (12.4%) |
| Unknown | 54 (<0.1%) | | 20 (<0.1%) | 18 (<0.1%) | | 5 (<0.1%) | | 11 (<0.1%) |
| **Prepandemic healthcare visits** |  | |  |  | |  | |  |
| Quantile 1 | 60994 (22.1%) | | 23341 (22.3%) | 9119 (9.8%) | | 5652 (21.9%) | | 22882 (43.3%) |
| Quantile 2 | 69544 (25.2%) | | 29895 (28.5%) | 16202 (17.4%) | | 7479 (29.0%) | | 15968 (30.2%) |
| Quantile 3 | 68114 (24.6%) | | 27718 (26.5%) | 23785 (25.6%) | | 7085 (27.5%) | | 9526 (18.0%) |
| Quantile 4 | 77731 (28.1%) | | 23792 (22.7%) | 43910 (47.2%) | | 5546 (21.5%) | | 4483 (8.5%) |
| **Number of chronic conditions** |  | |  |  | |  | |  |
| 2 | 50816 (18.4%) | | 19849 (18.9%) | 11861 (12.8%) | | 4975 (19.3%) | | 14131 (26.7%) |
| 3 | 66904 (24.2%) | | 26582 (25.4%) | 18495 (19.9%) | | 6635 (25.7%) | | 15192 (28.7%) |
| 4 | 59346 (21.5%) | | 23181 (22.1%) | 19515 (21.0%) | | 5673 (22.0%) | | 10977 (20.8%) |
| 5+ | 99317 (35.9%) | | 35134 (33.5%) | 43145 (46.4%) | | 8479 (32.9%) | | 12559 (23.8%) |
| **Prepandemic Diagnoses** |  | |  |  | |  | |  |
| Arthritis | 112573 (40.7%) | | 40937 (39.1%) | 44986 (48.4%) | | 9705 (37.7%) | | 16945 (32.1%) |
| Asthma | 41951 (15.2%) | | 12652 (12.1%) | 18925 (20.3%) | | 4272 (16.6%) | | 6002 (11.5%) |
| Atrial fibrilation | 51480 (18.6%) | | 21289 (20.3%) | 18636 (20.0%) | | 3662 (14.2%) | | 7893 (14.9%) |
| Cancer* | 30011 (10.9%) | | 11408 (10.9%) | 11567 (12.4%) | | 2530 (9.8%) | | 4506 (8.5%) |
| CKD | 55284 (20.0%) | | 17359 (16.6%) | 23992 (25.8%) | | 5860 (22.7%) | | 8073 (15.3%) |
| COPD | 39768 (14.4%) | | 14535 (13.9%) | 16250 (17.5%) | | 3518 (13.7%) | | 5465 (10.3%) |
| Depression | 46133 (16.7%) | | 12970 (12.4%) | 22385 (24.1%) | | 5020 (19.5%) | | 5758 (10.9%) |
| Diabetes | 121430 (43.9%) | | 43359 (41.4%) | 44840 (48.2%) | | 11902 (46.2%) | | 21293 (40.3%) |
| Heart failure | 32980 (11.9%) | | 11928 (11.4%) | 13873 (14.9%) | | 2581 (10.0%) | | 4598 (8.7%) |
| Hyperlipidemia | 209593 (75.8%) | | 81285 (77.6%) | 71549 (76.9%) | | 18798 (73.0%) | | 37961 (71.8%) |
| Hypertension | 262125 (94.8%) | | 99677 (95.2%) | 88348 (95.0%) | | 24181 (93.9%) | | 49919 (94.4%) |
| IHD | 83339 (30.2%) | | 33842 (32.3%) | 29445 (31.7%) | | 6300 (24.5%) | | 13752 (26.0%) |
| Osteoporosis | 32535 (11.8%) | | 12157 (11.6%) | 13150 (14.1%) | | 2810 (10.9%) | | 4418 (8.4%) |
| Stroke | 21228 (7.7%) | | 7368 (7.0%) | 8510 (9.2%) | | 1918 (7.4%) | | 3432 (6.5%) |
|  |  | |  |  | |  | |  |
| ADRD = Alzheimer's and related dementias, CKD = Chronic kidney disease, COPD = Chronic obstructive pulmonary disease, IHD = Ischemic heart disease | | | | | | | | |
| * Cancer = breast, colorectal, lung, prostate | | | |  | |  | |  |
| * Total: Patients missing both A1c & SBP measures were excluded (n=20504) | | | | | | | |  |
| ^  Controlled: No systolic blood pressure (SBP) not lower than 140 mmHg at last two visits prior to 3/7/20 and HbA1c not lower than 8.0% at last two visits prior to 3/7/20 | | | | | | | | |
| ǂ  Moderately Uncontrolled: Either at least one SBP that was 140-159 mmHg at last two visits prior to 3/7/20 or at least one HbA1c was 8.0-8.9% at last two visits prior to 3/7/20 | | | | | | | | |
| ꝉ Poorly Controlled: Either SBP not lower than 140 mmHg and HbA1c higher than 8.0%, or SBP higher than 160 mmHg, or HbA1c higher than 9.0%  Numbers in bold are reporting notable differences in subgroups compared to the total cohort. | | | | | | | | |
|  |  | |  |  | |  | |  |
| **Supplement Table 2** | | | | | |  | |  |
|  | | **Pre-Pandemic Control Status** | | | | | | |
|  | | **Controlled**  **(N=146077, 52.9%) ꝉ** | | | **Moderate       (N=86762, 31.4%) ꝉ** | | **Poor               (N =43449, 15.7%) ꝉ** | |
| **Characteristic** | |  | | |  | |  | |
| **Age** | |  | | |  | |  | |
| 50-64 | | -- | | | -- | | -- | |
| 65-74 | | 1.02 [0.99, 1.05] | | | 1.03 [0.98, 1.07] | | 1.08 [1.02, 1.16] | |
| 75-84 | | 1.07 [1.03, 1.11] | | | 1.08 [1.03, 1.13] | | 1.21 [1.13, 1.30] | |
| 85+ | | 1.22 [1.15, 1.29] | | | 1.15 [1.07, 1.23] | | 1.41 [1.28, 1.54] | |
|  | |  | | |  | |  | |
| **Sex** | |  | | |  | |  | |
| Male | | -- | | | -- | | -- | |
| Female | | 0.99 [0.96, 1.02] | | | 0.99 [0.96, 1.03] | | 1.00 [0.95, 1.06] | |
|  | |  | | |  | |  | |
| **Race/Ethnicity** | |  | | |  | |  | |
| White | | -- | | | -- | | -- | |
| Asian | | 1.21 [1.13, 1.29] | | | 1.16 [1.05, 1.28] | | 1.09 [0.94, 1.27] | |
| Black | | 0.99 [0.95, 1.04] | | | 0.96 [0.91, 1.01] | | 0.91 [0.84, 0.98] | |
| Hispanic | | 1.03 [0.99, 1.07] | | | 0.98 [0.93, 1.04] | | 0.90 [0.83, 0.97] | |
| Other/Unknown | | 1.18 [1.14, 1.23] | | | 1.13 [1.08, 1.19] | | 1.07 [0.99, 1.15] | |
|  | |  | | |  | |  | |
| **Neighborhood Poverty** | |  | | |  | |  | |
| Low | | -- | | | -- | | -- | |
| Medium | | 1.14 [1.10, 1.18] | | | 1.17 [1.12, 1.23] | | 1.21 [1.12, 1.30] | |
| High | | 1.13 [1.08, 1.18] | | | 1.13 [1.06, 1.20] | | 1.15 [1.05, 1.25] | |
| Very High | | 1.09 [1.03, 1.15] | | | 1.08 [1.01, 1.16] | | 1.15 [1.04, 1.26] | |
|  | |  | | |  | |  | |
| **COVID Infection** | | 0.12 [0.11, 0.13] | | | 0.12 [0.11, 0.14] | | 0.15 [0.13, 0.17] | |
|  | |  | | |  | |  | |
| **Pre-Pandemic healthcare visits** | |  | | |  | |  | |
| Q1 | | 8.76 [8.33, 9.21] | | | 8.56 [8.02, 9.14] | | 8.23 [7.53, 9.01] | |
| Q2 | | 4.34 [4.12, 4.56] | | | 4.48 [4.20, 4.78] | | 4.44 [4.07, 4.86] | |
| Q3 | | 2.48 [2.36, 2.62] | | | 2.47 [2.31, 2.64] | | 2.49 [2.28, 2.73] | |
| Q4 | | -- | | | -- | | -- | |
|  | |  | | |  | |  | |
| **Number of chronic conditions** | |  | | |  | |  | |
| 2 | | -- | | | -- | | -- | |
| 3 | | 0.90 [0.87, 0.94] | | | 0.89 [0.85, 0.94] | | 0.87 [0.81, 0.94] | |
| 4 | | 0.83 [0.79, 0.86] | | | 0.84 [0.79, 0.89] | | 0.87 [0.81, 0.95] | |
| 5+ | | 0.78 [0.75, 0.82] | | | 0.80 [0.76, 0.84] | | 0.79 [0.73, 0.85] | |
| **Supplement Table 3**   \| **Characteristic** \| Sensitivity Analysis:  Adjusted Model Predicting New Disruption in Acute Pandemic Period Only  (n=276288) \| Main Analysis:  Adjusted Model  (n=276288) \| \| --- \| --- \| --- \| \| **Age** \|  \|  \| \| 50-64 \| -- \| -- \| \| 65-74 \| 1.03 [1.01, 1.06] \| 1.03 [1.01, 1.06] \| \| 75-84 \| 1.14 [1.10, 1.17] \| 1.10 [1.06, 1.13] \| \| 85+ \| 1.30 [1.24, 1.35] \| 1.23 [1.18, 1.28] \| \|  \|  \|  \| \| **Sex** \|  \|  \| \| Male \| -- \| -- \| \| Female \| 1.01 [0.98, 1.03] \| 0.99 [0.97, 1.01] \| \|  \|  \|  \| \| **Race/Ethnicity** \|  \|  \| \| White \| -- \| -- \| \| Asian \| 1.17 [1.10, 1.24] \| 1.18 [1.12, 1.24] \| \| Black \| 0.96 [0.93, 0.99] \| 0.97 [0.94, 1.00] \| \| Hispanic \| 1.00 [0.97, 1.04] \| 0.99 [0.96, 1.02] \| \| Other/Unknown \| 1.11 [1.07, 1.14] \| 1.15 [1.12, 1.19] \| \|  \|  \|  \| \| **Neighborhood Poverty** \|  \|  \| \| Low \| -- \| -- \| \| Medium \| 1.12 [1.09, 1.16] \| 1.16 [1.13, 1.19] \| \| High \| 1.11 [1.07, 1.15] \| 1.13 [1.09, 1.17] \| \| Very High \| 1.07 [1.03, 1.12] \| 1.09 [1.05, 1.14] \| \|  \|  \|  \| \| **COVID Infection** \| 0.13 [0.12, 0.14] \| 0.12 [0.12, 0.13] \| \|  \|  \|  \| \| **Pre-Pandemic healthcare visits** \|  \|  \| \| Q1 \| 3.34 [3.21, 3.47] \| 8.61 [8.30, 8.93] \| \| Q2 \| 3.46 [3.34, 3.60] \| 4.39 [4.24, 4.56] \| \| Q3 \| 2.31 [2.22, 2.41] \| 2.48 [2.39, 2.58] \| \| Q4 \| -- \| -- \| \|  \|  \|  \| \| **Number of chronic conditions** \|  \|  \| \| 2 \| -- \| -- \| \| 3 \| 0.96 [0.93, 0.99] \| 0.89 [0.87, 0.92] \| \| 4 \| 0.93 [0.90, 0.97] \| 0.84 [0.81, 0.86] \| \| 5+ \| 0.87 [0.84, 0.90] \| 0.79 [0.76, 0.81] \| \|  \|  \|  \| \| **Health status** \|  \|  \| \| Controlled \| -- \| -- \| \| Moderate Uncontrolled \| 1.04 [1.02, 1.07] \| 1.00 [0.98, 1.03] \| \| Poorly controlled \| 1.07 [1.03, 1.10] \| 1.02 [0.99, 1.05] \| | | | | | | | | |
|  | | |  |  | |  | |  |
|  | | | |  | |  | |  |
